# Alternative promoter usage during organ development

**DOI:** 10.1371/journal.pgen.1011635

**Published:** 2025-03-28

**Authors:** Jiang Tan, Yidan Sun

**Affiliations:** 1 Department of Genetics, Washington University School of Medicine, St. Louis, Missouri, United States of America; 2 McDonnell Genome Institute, Washington University School of Medicine, St. Louis, Missouri, United States of America; 3 Institute for Informatics, Data Science and Biostatistics (I2DB), Washington University School of Medicine, St. Louis, Missouri, United States of America; Indiana University Purdue University at Indianapolis, UNITED STATES OF AMERICA

## Abstract

Dynamic gene expression is crucial for mammalian organ development, influencing organ-specific functions and responses. A significant number of mammalian protein-coding genes are regulated by multiple distinct promoters, suggesting that the choice of promoter is as critical as its transcriptional output. However, the role of alternative promoters in organ development remains largely unexplored. In this study, we utilized RNA-seq data from 313 mouse samples across various developmental stages in seven major organs to identify active promoters. Our analyses revealed between 967 and 3,237 developmentally dynamic promoters (DDPs) in each organ. These DDPs encompass not only major promoters with the highest activity within a gene but also alternative promoters with lower activity, which are often overlooked in traditional gene-level analyses. Notably, we found that alternative DDPs can be independently regulated compared to their major counterparts, suggesting the involvement of unique transcriptional regulatory mechanisms. Furthermore, we observed that increased alternative promoter usage plays a pivotal role in driving organ-specific functions and gene expression alterations. Our findings underscore the importance of alternative promoter usage in shaping organ identity and function, providing new insights into the regulatory complexity of organogenesis.

## Author summary

How do organs develop their unique identities and functions? While many studies focus on the activity of genes, our research takes a closer look at the “on switches” of genes—called promoters—that regulate how genes are turned on and off. Many genes have multiple promoters, but the role of these alternative promoters during organ development has been largely overlooked.

In this study, we analyzed data from 313 samples of developing mouse organs, spanning seven key tissues and multiple stages of development. We identified thousands of promoters whose activity changes dynamically over time. Importantly, these include not only the main promoter driving most gene activity but also alternative promoters, which have their own unique patterns of regulation. We discovered that these alternative promoters are often independently controlled and play a critical role in organ-specific functions, influencing how genes are expressed differently in the heart, brain, liver, and other organs.

Our findings provide a new perspective on how genes are regulated during organ development, revealing the intricate mechanisms behind organ-specific gene expression. This study offers valuable insights for understanding the complexity of organ formation and may help inform future research into developmental biology and genetic regulation.

## Introduction

Promoters, regions upstream of transcription start sites (TSSs), are essential elements in transcription regulation. They house the necessary regulatory components for transcription initiation and integrate signals from distal regulatory elements and epigenetic modifications that determine transcriptional output. In the mammalian genome, most protein-coding genes are regulated by multiple promoters, which drive the expression of different gene isoforms [1,2]. Unlike post-transcriptional regulation, such as alternative splicing, alternative promoter usage allows for pre-transcriptional regulation of gene isoform expression [3].

Promoter usage has been shown to play crucial roles in organ development. For example, changes in promoter usage of the transcription factor MITF have been found to regulate the development of the retina and retinal pigment epithelium during eye formation [4]. Genome-wide studies, utilizing markers like H3K4me3 (a histone modification marking active promoters) and 5′ end cap-analysis of gene expression (CAGE), have revealed differential promoter usage between pluripotent cells and differentiated organs in mammals [5]. However, while these insights offer valuable glimpses, the role of alternative promoters in organ development remains largely unexplored due to the scarcity of comprehensive datasets like H3K4me3 ChIP-seq or CAGE for most organ-related studies.

RNA sequencing (RNA-seq) has emerged as one of the most powerful and widely used tools for studying gene expression on a large scale, providing a nearly unbiased view of the transcriptome. Beyond measuring total gene expression, RNA-seq offers the potential to pinpoint active promoters by identifying transcript isoforms with distinct 5′ start sites, linked to different promoters. For instance, by analyzing RNA-seq data, we can distinguish between promoter-specific transcripts, allowing us to infer which promoters are active at various stages of development. Unlike techniques like H3K4me3 ChIP-seq or CAGE, which are often unavailable for many organ-related studies, RNA-seq is more accessible and widely applied. This makes RNA-seq an invaluable tool for mapping promoter activity across organs and developmental stages, revealing how alternative promoters contribute to the diversity of gene expression [6–8].

In this manuscript, we infer active promoters from bulk RNA-seq data across different developmental stages, covering multiple organs from early organogenesis to adulthood in mice. Our findings reveal that alternative promoters are frequently used to increase isoform diversity throughout organ development. While common genes across organs tend to exhibit promoter downregulation, organ-specific genes show increased promoter activity as development progresses. Our data suggest that the landscape of active promoters is highly dynamic, with organ-specific variation in promoter usage driving specialized functions. We propose that understanding which promoters are active at different developmental stages provides key insights into the genetic, transcriptional, and pathological processes underlying organ development.

## Results

### Identification of developmentally active promoters in 313 mouse RNA-seq samples

**Fig 1:**
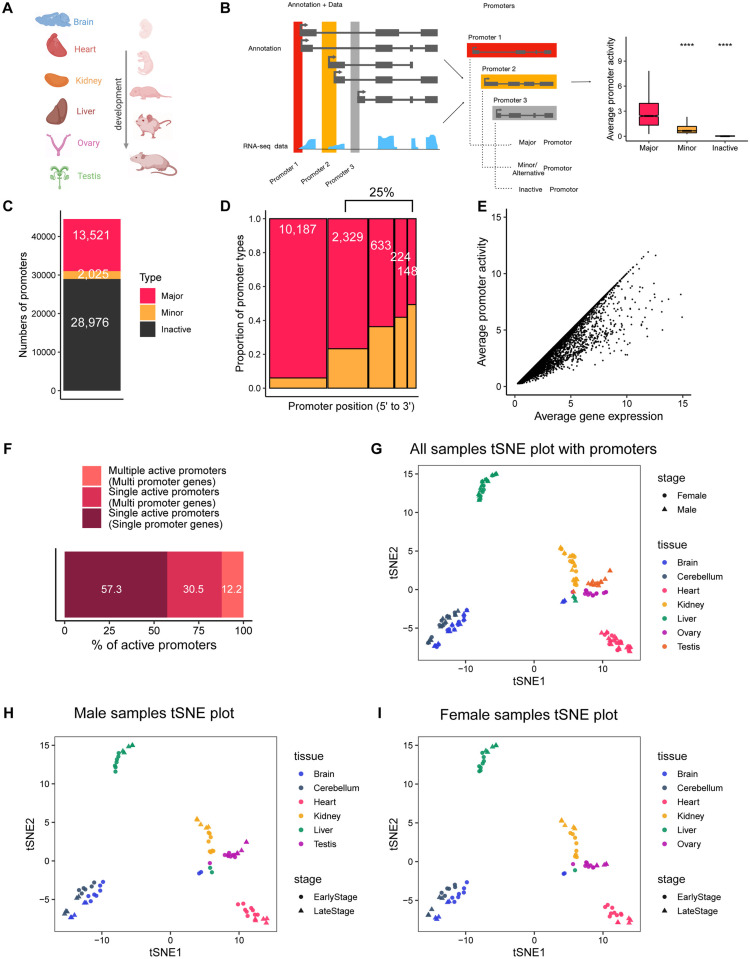
Identification of Developmentally Active Promoters in 313 Mouse RNA-seq Samples. (A) Schematic representation of the RNA-seq datasets used in this study, encompassing the developmental stages of seven organs. The diagram was created in BioRender. Sun, Y. (2025) https://BioRender.com/o54j718. (B) Overview of promoter-activity quantification using RNA-seq data. Isoforms regulated by the same promoter are grouped, and promoter activity is estimated using unique junction reads spanning the first intron of each isoform. (C) Classification of annotated promoters based on average promoter activity across all RNA-seq samples. Promoters are categorized into three groups: major promoters, the most active promoter of each gene; minor/alternative promoters, other active promoters of the gene; and inactive promoters, with an estimated activity of <0.25. (D) Distribution of major and minor/alternative promoters across transcription start sites (TSSs), ranked from 5′ to 3′, for multi-promoter genes with at least one active promoter. (E) Comparison between major promoter activity and total gene expression (sum of all promoters). A single promoter often does not fully represent the gene’s expression, as minor/alternative promoters contribute additional regulatory information. (F) Proportions of genes with a single promoter, a single active promoter, and multiple active promoters. (G-I) t-SNE plots based on the top 2,000 promoters with the highest variance in activity, displayed for all samples (G), male samples (H), and female samples (I).

To investigate the role of active promoters during mammalian organ development, we analyzed a comprehensive RNA-seq dataset covering the developmental stages of seven major organs—forebrain/cerebrum (hereafter referred to as “brain”), cerebellum, heart, kidney, liver, ovary, and testis—from early organogenesis to adulthood in mice [9–11] (Fig 1A). The objective was to identify an atlas of developmentally active promoters across these organs.

Using the definition of promoters as regulatory regions upstream of TSSs, we found that 24% of all annotated genes in mice have more than one promoter (S1A Fig). We compiled a set of 76,657 possible promoters based on Ensembl (version 91) annotations (S1 Table), assuming isoforms with identical or closely positioned TSSs are regulated by the same promoter [12,13]. Since the number of promoters is significantly smaller than the number of isoforms per gene, promoter activity estimation is computationally simplified, resulting in more robust inferences [14]. To minimize false positives, we excluded internal promoters that overlapped with non-first exons in certain isoforms (see Methods) [14]. In total, 44,522 promoters were retained for further analysis.

Promoter activity was quantified as the total expression initiated at each promoter using unique junction reads from the RNA-seq data (see Methods) [14]. For each gene, the promoter with the highest activity was designated as the “major promoter,” while promoters with lower activity were labeled as “minor promoters”, also referred to as “alternative promoter” (Fig 1B and S1 Table). Promoters that exhibited no expression were classified as “inactive promoters“ (Fig 1B).

Across all samples, we identified the major promoter for 13,521 genes (30.4%). Additionally, 2,025 minor/alternative promoters (4.5%) were found to be active, and 65.1% (28,976) of the promoters were classified as inactive (Fig 1C). Interestingly, while it is often assumed that the first promoter of a gene is the active one, our data revealed that 25% of major promoters were located downstream of the first TSS (Fig 1D), suggesting that the dominant promoter can occur at various positions within a gene.

Promoter activity also allowed us to assess the contribution of individual promoters to the overall gene expression profile [14]. Among expressed protein-coding genes, 12.2% had at least two active promoters, each contributing more than 10% to the gene’s overall expression (Fig 1E and 1F). These minor/alternative promoters, whose activity varies based on developmental context, are typically overlooked in standard gene-level expression analyses.

To further explore promoter activity, we performed a t-SNE analysis using the 2,000 most diverse promoters across the organs. We found that promoter activity clustered by tissue type rather than by gender (Fig 1G, 1H, and 1I). This mirrors findings from gene expression analyses (S1B, S1C, and S1D Fig), indicating that promoter activity can serve as a proxy for gene expression in mice.

Collectively, these results demonstrate that promoter activity provides additional layers of information to genome annotations and complements standard gene-level analyses, despite its general resemblance to gene expression patterns at a global level. These findings are consistent with previous studies in human samples [14].

### Characterization of developmentally dynamic promoters (DDPs)

Interestingly, promoter activity showed distinct patterns between early and late developmental stages in both male and female samples (Fig 1H and 1I), suggesting that the regulation of promoter activity may play a crucial role in organ development. To explore this further, we aimed to identify promoters with significant temporal activity changes across developmental stages in each organ using a regression approach from the maSigPro package [15], which we termed *developmentally dynamic promoters* (DDPs) (S1 and S2 Tables; see Methods). Across the seven organs, we identified between 967 to 3,237 DDPs, indicating the numbers of DDPs vary across organs.

**Fig 2:**
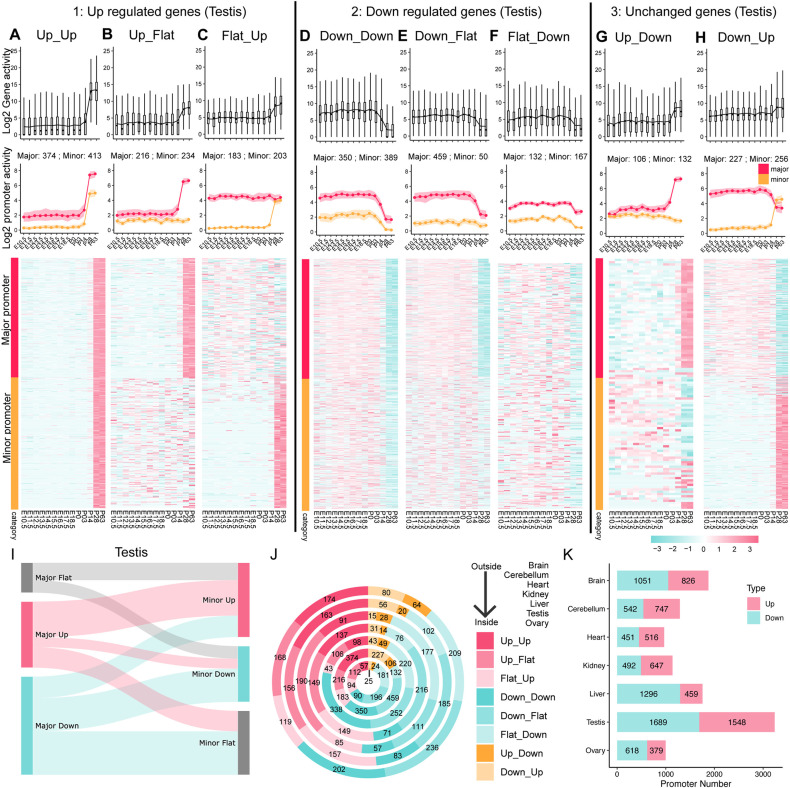
Characterization of Developmentally Dynamic Promoters (DDPs). (A-H) Developmental dynamics analysis of testis RNA-seq data. DDP associated genes are classified based on total gene expression changes into three categories: upregulated, downregulated, and unchanged (or mildly changed). Additionally, major and minor/alternative promoter pairs exhibit eight distinct patterns over developmental time (see Methods). For upregulated genes: (A) Up_Up – Both major and minor/alternative promoters show increased activity. (B) Up_Flat – Major promoter activity increases while minor/alternative promoter activity remains unchanged. (C) Flat_Up – Opposite of Up_Flat, with minor/alternative promoter activity increasing and major promoter activity unchanged. For downregulated genes: (D) Down_Down – Both major and minor/alternative promoters show decreased activity. (E) Down_Flat – Major promoter activity decreases while minor/alternative promoter activity remains unchanged. (F) Flat_Down – Opposite of Down_Flat, with minor/alternative promoter activity decreasing and major promoter activity unchanged. For unchanged (or mildly changed) genes: (G) Up_Down – Major promoter activity increases while minor/alternative promoter activity decreases. (H) Down_Up – Opposite of Up_Down, with major promoter activity decreasing and minor/alternative promoter activity increasing. For each category, the upper panel shows the mean total gene activity (sum of all promoter activities) across developmental stages. The middle panel shows the mean major (red) and minor/alternative (orange) promoter activity changes over developmental stages, with shading representing 1,000 bootstraps. The bottom panel presents heatmaps illustrating each major and minor/alternative promoter activity across developmental stages. (I) Sankey plot displaying the proportions of the eight categories (A-H). The left panel includes all major promoters with unchanged, upregulated, and downregulated across developmental stages, while the right panel shows minor/alternative promoters with the same classification. (J) Donut plot summarizing the number of genes in each of the eight categories (A-H) for each organ. Note that the number on the plot refers to the gene number, with major and minor promoters considered as a pair. Thus, the counts equal the number of major promoters shown in (A-H), as there is always one major promoter per gene. (K) Bar chart showing the number of developmentally upregulated (red) and downregulated (blue) promoters across organs. Note that the sum of up and down major and minor promoters in panels a–h aligns closely with the total number shown in panel k, but minor discrepancies may arise due to cases where only one of the alternative promoters is upregulated while the other remains flat from the same gene. Specifically, panels a–h classify promoters based on total gene expression levels, showing all major and minor promoters associated with a gene. In this case, one major promoter may correspond to two alternative promoters. While the major promoter is always upregulated, most alternative promoters are also upregulated; however, in some cases, only one alternative promoter is upregulated while the other remains unchanged.

Surprisingly, DDPs encompass not only major promoters but also minor/alternative promoters (Figs 2 and S2–S7 Figs), which is often overlooked in traditional analyses. More interestingly, not all DDP corresponding genes exhibited developmentally dynamic expression at the gene level (Fig 2G and H; upper panel), highlighting how promoter-specific analysis allowed us to identify a previously unrecognized class of developmentally dynamic genes.

**Fig 3:**
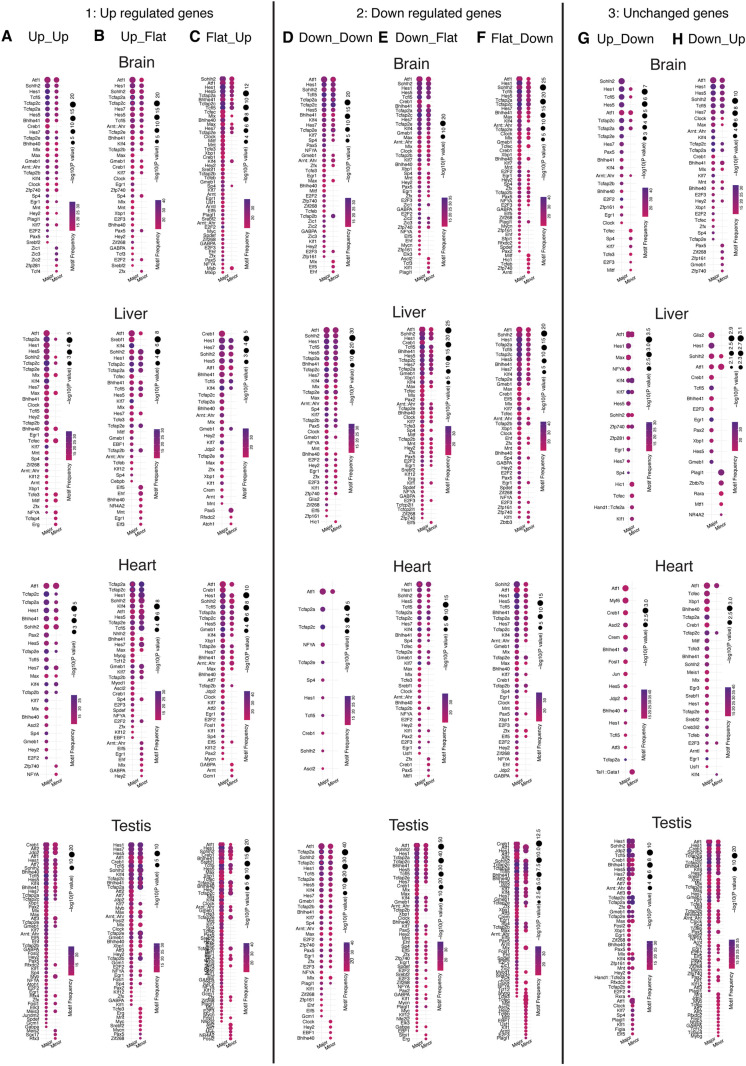
Independent Regulation of Alternative DDPs. (A-H) Enrichment analysis of transcription factor (TF) motifs for major and minor/alternative promoters across different categories in each organ. The dot plot visualization displays key enrichment results, with dot size representing the -log10(p-value), indicating the statistical significance of the TF motif enrichment, and dot color representing the motif occurrence proportion, reflecting the frequency of the corresponding TF motif in the analyzed promoter regions.

We next categorized the DDPs based on their patterns of promoter activity. First, we classified the DDP corresponding genes into *upregulated*, *downregulated*, and *unchanged* (or *mildly changed*) categories, depending on the total gene expression changes over developmental time. Interestingly, the paired activities of major and minor/alternative promoters revealed eight distinct patterns across developmental stages (see Methods). Taking testis as an example (Fig 2A-H, red represents the major promoter, orange represents the minor/alternative promoter and black represents the gene), these patterns are as follows:

(1) For upregulated genes: *Up_Up* - Both major and minor/alternative promoters’ activity are upregulated (Fig 2A); *Up_Flat* - Only major promoter’s activity is upregulated and minor/alternative promoter’s activity remains unchanged (Fig 2B); *Flat_Up* - Contrary to *Up_Flat,* only minor/alternative promoter’s activity is upregulated and major promoter’s activity remains unchanged (Fig 2C);
(2) For downregulated genes: *Down_Down* - Both major and minor/alternative promoters’ activity are downregulated (Fig 2D); *Down_Flat* - Only major promoter’s activity is downregulated and minor/alternative promoter’s activity remains unchanged (Fig 2E); *Flat_Down*- Contrary to *Down_Flat,* only minor/alternative promoter’s activity is downregulated and major promoter’s activity remains unchanged (Fig 2F);
(3) For unchanged (or mild changed) genes: *Up_Down* - major promoter’s activity is upregulated, while minor/alternative promoter’s activity is downregulated (Fig 2G); *Down_Up* - Contrary to *Up_Down,* major promoter’s activity is downregulated, while minor/alternative promoter’s activity is upregulated (Fig 2H).

Similar patterns were observed in the other six organs analyzed (S2–S7 Figs).

We further compared the promoter activity of DDPs to the flat (developmentally non-changed) promoters in each organ. Although the mean value of DDPs during developmental stages showed a significant change compared to flat promoters, the difference was not prominent. Similarly, the median activity value of DDPs mirrored the results observed with the mean value. However, the maximum activity value of DDPs was dramatically higher than that of flat promoters. These findings suggest that flat promoters primarily contribute to the maintenance of baseline gene expression levels throughout development, providing consistent expression. In contrast, DDPs exhibit sharper peaks of activity, reflecting their critical roles in mediating developmental transitions. This distinction indicates the unique functional contributions of DDPs in shaping organ development.

### Independent regulation of alternative DDPs

Through our categorization, we observed that a moderate portion of minor/alternative promoters are co-regulated with their corresponding major promoters in each tissue, showing either concurrent upregulation (Up_Up) or downregulation (Down_Down) (Fig 2I and 2J). However, a subset of minor/alternative promoters exhibited negative co-regulation with their major counterparts, where changes in promoter activity occur in opposite directions, maintaining a stable overall gene expression level (Up_Down or Down_Up) (Figs 2G–J, S2–S7G, and S7H). More Interestingly, around 50% of minor/alternative promoters demonstrated unrelated regulation compared to their major promoters, displaying patterns such as Up_Flat, Flat_Up, Down_Flat, and Flat_Down (Figs 2B, 2C, 2D, 2F, 2I, 2J, S2–S7B, S7C, S7D, and S7F). These observations suggest that alternative promoters are independently regulated by distinct transcription factors or cofactors during organ development, rather than being governed by the same regulatory mechanisms as their major counterparts.

We further performed transcription factor (TF) motif enrichment analysis for each category in each tissue (Figs 3 and S9 and S3 Table). For the Up_Down or Down_Up categories, there are clear differences in enriched TFs between major and minor promoters, as expected (Figs 3G and H and S9G and H), indicating they are regulated by different TFs. This may explain why major and minor/alternative promoters in these categories exhibit changes in opposite directions during development. Similarly, for categories such as Up_Flat, Flat_Up, Down_Flat, and Flat_Down—where minor/alternative promoters display distinct patterns compared to their major counterparts—a notable difference in enriched TFs was also observed (Figs 3B, 3C, 3E, 3F, S9B, S9C, S9E, and S9F). This difference was particularly prominent in tissues such as the brain, kidney, testis, and ovary for Flat_Down (Figs 3F and S9F), and the heart, kidney, liver, testis, and ovary for Flat_Up (Figs 3C and S9C). Notably, a considerable number of enriched TFs are shared between major and minor/alternative promoters in the above categories, indicating that minor/alternative promoters may be not only regulated by distinct TFs but also by different cofactors, which assist in the binding and activity of TFs[16].

Lastly, for the Up_Up and Down_Down categories, which exhibit concurrent upregulation or downregulation, the enriched TFs are mostly shared between major and minor/alternative promoters. However, some differences still exist (Figs 3A, 3D, S9A, and S9D). This further supports the notion that alternative promoters are regulated by different transcriptional machinery compared to their major counterparts.

In summary, our findings highlight that the dynamic activity patterns of alternative promoters can differ significantly from their major promoters. Furthermore, transcription factors enriched at alternative promoters are often distinct from their major counterparts. Together, these results suggest that alternative promoters are independently regulated during organ development.

### Organ-specific patterns of alternative DDPs

**Fig 4:**
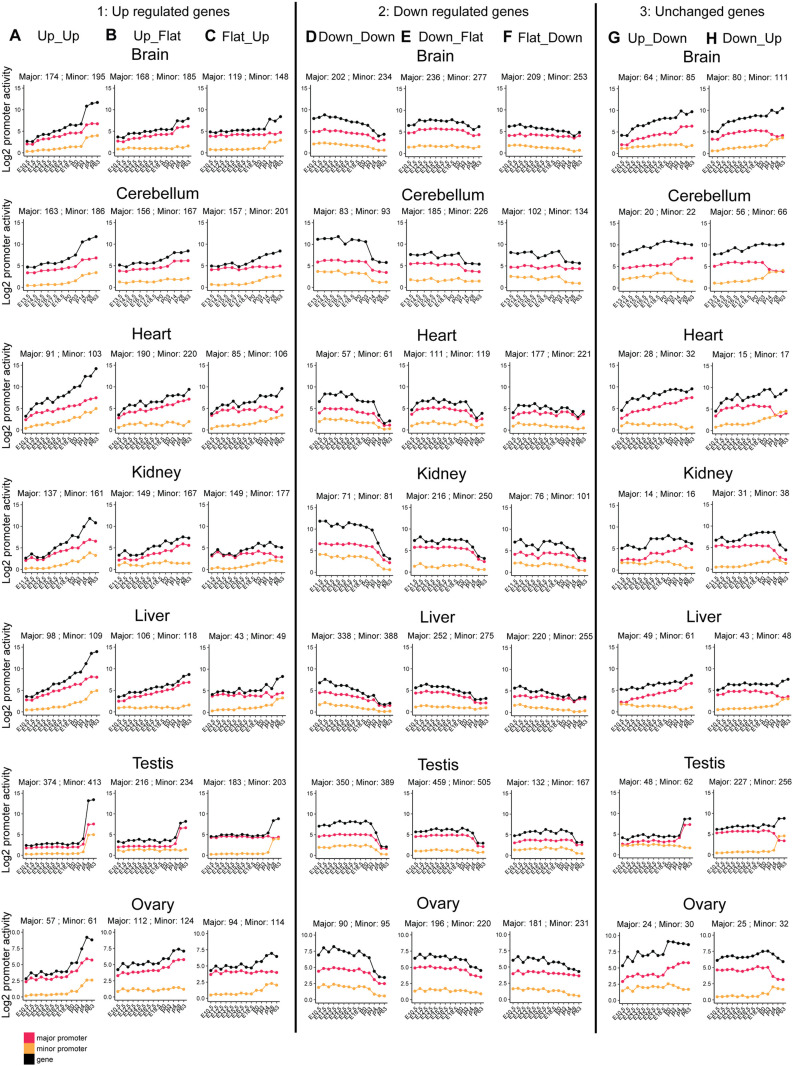
Organ-Specific Patterns of Alternative DDPs. (A-H) Developmental dynamic promoter analysis across all organs. In each organ, major and minor/alternative promoter pairs exhibit eight distinct patterns over developmental time (consistent with Figs 2A-H and S2–S7 Fig, middle panels). In each plot, the red line represents major promoter activity, the yellow line represents minor/alternative promoter activity, and the black line represents overall gene activity.

Given that alternative DDPs are independently regulated during organ development, we next explored their dynamic pattern across organs during development. We analyzed downregulated minor/alternative promoters (activated earlier in development) and upregulated promoters (activated later in development) separately (Fig 4). A common pattern is observed for the downregulated promoters. They showed the most pronounced decrease around the time of birth across all organs (Figs 4D-G, 2D-G, S2–S7D and S7G).

In contrast, we found the patterns of upregulated minor/alternative promoters differ among organs. For those in the heart, kidney, and liver, we observed a gradual increase in minor/alternative promoter activity throughout development, lacking a distinct surge at any specific time point (Figs 4A–C, 4H, S4–S6A, S6C, and S6H).

Conversely, in the brain and cerebellum, there is a sharp increase in minor/alternative promoter activity starting at postnatal day 3 (P3), corresponding to critical periods of brain development (Figs 4A–C, 4H, S2, S3, S7A–C, and S7H). In the testis, the most significant upregulation occurs after postnatal day 14 (P14), aligning with puberty and the extensive developmental changes associated with that stage (Figs 2A–C, 2H, 3A–C, and 3H).

These results underscore the functional relevance of alternative promoter usage in organ development, as corresponding changed periods are closely linked to the establishment of organ identity and the transition to mature organ-specific functions.

### Alternative promoter usage drives organ specialized functions

**Fig 5:**
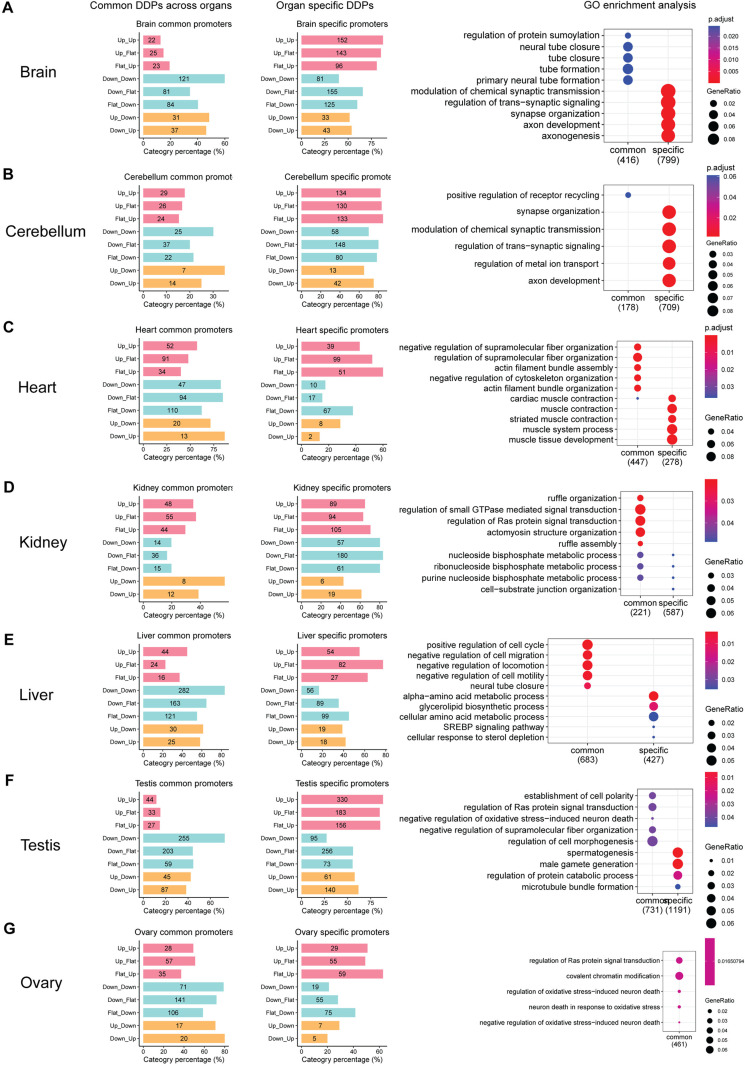
Alternative Promoter Usage Drives Organ Specialized Functions. (A-G) Organ-specific developmental dynamic promoter analysis. Left and middle panels: Bar plots showing the proportion of developmentally dynamic promoters that are common across organs versus those that are organ-specific within each category for each tissue, as shown in Fig 4. The exact number is shown on the barplot. Right panel: Gene ontology (GO) enrichment analysis of developmentally dynamic promoters that are common across organs and those that are organ-specific in each tissue. The size of the black dots indicates the ratio of genes, and the adjusted p-value is represented by a color gradient from red to blue. Note: Ovary-specific DDPs did not yield significant GO enrichment results, likely due to the small number of genes.

As the patterns of alternative DDPs vary among organs, we compared the DDPs across multiple tissues and identified both common and organ-specific DDPs. Interestingly, common DDPs, shared across at least 2 organs (see Methods for details), are more prone to be downregulated promoters (activated earlier in development) (Fig 5; left panel). Through the GO enrichment analysis, we found that they are enriched in more basic functions, such as embryo development and cell cycle related functions (Fig 5 right panel and S4 Table). As expected, many GO terms are shared across tissues (S10A Fig and S4 Table).

In contrast, organ-specific DDPs, only observed in one organ but not the others (see Methods for details), are more prone to be upregulated promoters (activated later in development) (Fig 5 middle panel). We further performed GO enrichment analysis, the organ-specific activated promoters are enriched in organ specialized functions, with small overlap between tissues (S10B Fig and S4 Table). For example, brain and cerebellum specific promoters are enriched in neuron developmental functions, such as synapse organization and axon development (Fig 5A and 5B; right panel). Heart specific promoters are enriched in cardiac muscle contraction and muscle development functions (Fig 5C; right panel). Testis specific promoters are enriched in spermatogenesis and male gamete generation (Fig 5F; right panel).

Collectively, our analysis demonstrates that increased alternative promoter usage in later developmental stages is enriched in organ-specific functions, highlighting its crucial role in organ specialization and the generation of organ-specific phenotypes.

### Alternative promoter usage as a key driver of organ-specific gene expression alterations

Previous studies have shown that the transcriptomes of different organs exhibit strong similarities during early developmental stages but diverge into organ-specific developmental programs as they mature [9]. Since increased usage of alternative promoters is linked to organ-specific functions, we explored the relationship between alternative promoter usage and gene expression during development. Our previous analysis of eight distinct promoter activity patterns revealed that overall changes in gene expression are not solely driven by major promoters; minor/alternative promoters also play a crucial role (Figs 2, 4, and S2–S7). For example, in patterns such as Up_Up, Flat_Up, Flat_Down, and Down_Down (Figs 2A, 2C, 2D, 2F, S2–S7A, S7C, S7D, and S7F), alternative promoter usage (orange line) significantly contributes to gene expression alterations during organ development.

**Fig 6:**
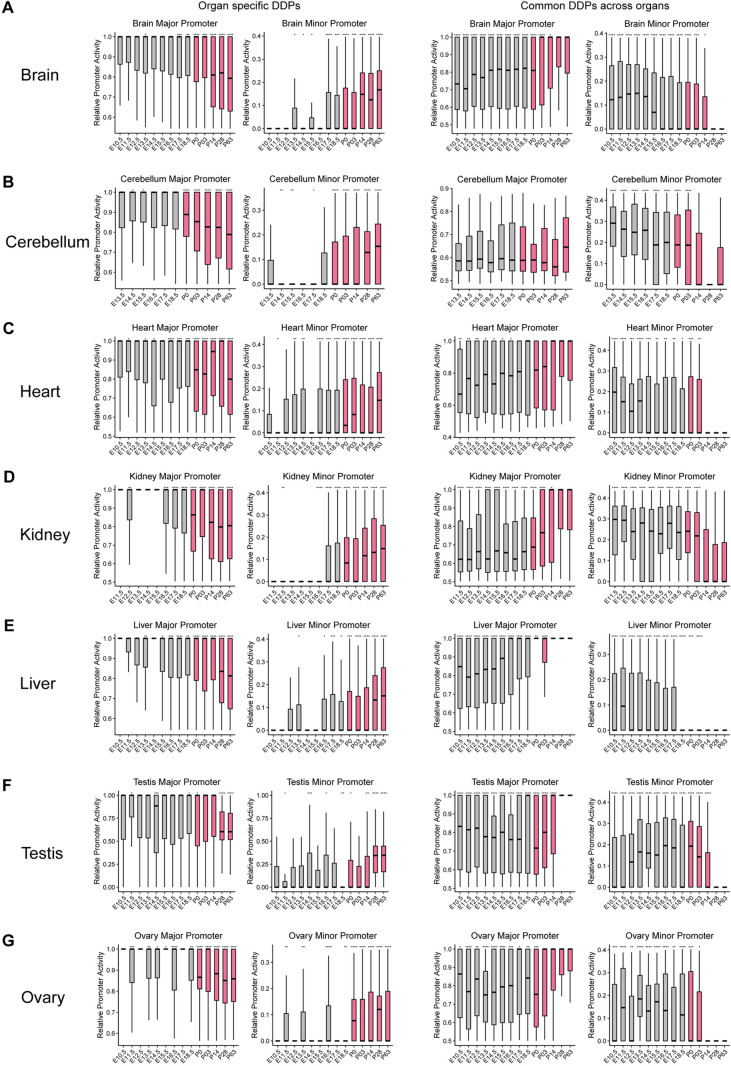
Alternative Promoter Usage as a Key Driver of Organ-Specific Gene Expression Alterations. (A-G) Relative activity profiles of developmentally dynamic promoters (DDPs) that are organ-specific (left) and common across organs (right) for each tissue. Organ-specific promoters exhibit increased minor/alternative promoter activity during the later stages of development (pink represents later stages), while common promoters display the opposite trend.

To further investigate this association, we calculated the relative contributions of major and minor/alternative promoters to total activity. For organ-specific expressed genes, we observed an increase in minor/alternative promoter usage in the later stages of development, while major promoter usage decreased (Fig 6; left panel). Conversely, for common genes expressed across multiple organs, minor/alternative promoter usage decreased in the later stages, and major promoter usage increased (Fig 6; right panel). This trend was consistent across all seven organs, suggesting that the increase in organ-specific gene expression during development may be primarily driven by alternative promoter usage, rather than just an amplification of major promoter activity.

Beyond their role in transcriptional regulation, we also explored whether alternative promoter usage could drive the production of distinct protein isoforms. Hence, we examined the coding potential of transcripts initiated from alternative promoters. Our analysis revealed that approximately 60-90% of transcripts initiated from alternative promoters are protein-coding (S11 Fig). This finding suggests that a substantial majority of alternative promoters may exert functional effects beyond transcriptional regulation, potentially influencing protein diversity and function. These results underscore the significance of alternative promoter usage in modulating gene expression and expanding the proteomic landscape.

Overall, our findings indicate that alternative promoters are key contributors to transcriptional diversity during organ development—a factor often overlooked in traditional gene-level analyses. More importantly, the increased use of alternative promoters may drive organ-specific gene expression changes, leading to specialized organ functions.

## Discussion

Through re-exploring the RNA-seq data from early organogenesis to adulthood across seven major organs, our study highlights the significant role of alternative promoter usage in mammalian organ development, which is often overlooked in traditional gene-level analyses. This study marks the first systematic examination of promoter usage across mammalian development, with direct comparisons between organs. By identifying developmentally dynamic promoters (DDPs) across major organs, we provide a complete study of promoter activity in mouse organ development. Our detailed annotation of promoter activity across tissues offers a valuable resource for the research community, providing insights into the regulatory complexity of organogenesis. More importantly, we demonstrate that many alternative promoters are independently regulated and contribute to organ-specific gene expression. These findings reveal the intricate transcriptional mechanisms that drive organ identity and function, emphasizing the regulatory complexity of organogenesis and suggesting new avenues for understanding gene regulation in development.

In this study, we demonstrate that many alternative promoters exhibit dynamic developmental patterns and enriched TFs distinct from their major counterparts, suggesting they are regulated by different TFs and cofactors. Given that alternative promoter usage drives organ-specific functions, our findings raise the possibility that TFs and cofactors regulating these promoters are crucial for organogenesis and may have been previously overlooked. Traditional TF and cofactor binding studies have typically focused on major or first promoters, leaving alternative promoters underexplored [14,16]. As a result, the roles of TFs and cofactors involved in regulating alternative promoters may have been underestimated. Thus future studies should focus on newly identified TFs from alternative promoters and their functions. This will help us gain a more comprehensive understanding of the regulatory networks driving organ-specific gene expression and function, uncovering new regulatory mechanisms essential for organogenesis.

Previous studies have shown that, due to changes in pleiotropy during development, different organs are most similar early on and become increasingly distinct as development progresses [9]. In our study, we found that major promoters tend to be active across multiple organs and stages in early development, rendering them more refractory to change. As organs differentiate and mature, the use of alternative promoters increases, leading to greater diversity in promoter usage for individual genes. This finding suggests a novel form of gene dosage regulation: instead of simply upregulating the original promoter, the organism activates a new promoter. This strategy appears to offer a more flexible and efficient way for organs to regulate gene dosage in response to different developmental contexts. It may also help explain the increased divergence in gene expression across species as development advances, a pattern observed in mammalian limb development [17], whole embryos [18] and other organs [9]. Future studies should focus on exploring the mechanism of alternative promoter usage at both genetic and epigenetic level. Investigating these mechanisms may lead to novel therapeutic strategies for developmental diseases. On the other hand, the functional consequences of this regulation, including its impact on protein isoform diversity and potential tissue-specific protein function, warrant further investigation. Understanding how these mechanisms operate during development may provide insights into the evolution of organ-specific functions and the molecular underpinnings of organogenesis.

Our study is limited to bulk RNA-seq data, and a key challenge moving forward will be to disentangle the precise contributions of cell composition and cell-type-specific promoter activity changes to the developmental dynamic promoter patterns we observed. Future research should complement our findings with single-cell transcriptomic and epigenomic datasets. Such approaches will provide a more detailed understanding of the genetic and developmental mechanisms underlying organ development and the emergence of organ-specific phenotypes.

## Materials and methods

### RNA-seq pre-processing

The data was processed via the snakePipes mRNA-seq pipeline (v2.1.2) [19]. Adapters and low quality bases (< Q20) were removed using TrimGalore (v0.6.5) (https://github.com/FelixKrueger/TrimGalore). The trimmed reads were aligned using STAR (v2.7.4) [20] to Ensembl mouse genome (version 91) with ‘2-pass mode’. STAR junction files were used for the following promoter activity analysis.

### Identification of promoters from annotations

Ensembl (version 91) mouse annotations were used to identify TSSs (defined as the start of the first exon) for all annotated transcripts with R package “proActiv” [14]. As transcripts with identical or very close TSSs are more prone to be regulated by the same promoter, we defined promoters using the TSSs from overlapping first exons and 5′ most TSS was defined as the TSS for each promoter. This approach enabled us to map promoters to their associated transcripts and genes. These promoter mappings were subsequently used to quantify promoter activity for each gene in each sample during downstream analyses.

### Promoter activity estimation

We quantified both absolute and relative promoter activities for uniquely identifiable promoters in each sample using the junction read counts method, implemented through the R package “proActiv” [14]. This approach estimates promoter activity based on RNA-seq data by leveraging read counts spanning exon-exon junctions that map to the first introns of transcripts associated with each promoter.

- Absolute Promoter Activity: For each sample, the absolute promoter activity was calculated as the log2-transformed total junction read counts aligning to the first introns. To account for differences in sequencing depth, we applied size factors computed using the DESeq2 (v1.20.0) package [21] to normalize these counts across all samples. The normalized counts were then log2-transformed and used as the measure of promoter activity for downstream analyses.
- Gene Expression: Gene expression for each sample was determined as the sum of the absolute promoter activities across all promoters associated with that gene.
- Relative Promoter Activity: To calculate the relative promoter activity, we divided each promoter’s normalized activity by the total gene expression for that sample. This allowed us to assess the contribution of individual promoters to the overall gene expression in a relative context.

### Definition of major, minor/alternative, inactive promoters

We categorized the promoters into three distinct groups based on their absolute activity levels: major, minor/alternative, and inactive promoters. The promoter with the highest average activity for each gene across all RNA-seq samples was designated as the major promoter. Promoters with an average activity below 0.25 were classified as inactive, while the remaining promoters with intermediate activity were categorized as minor/alternative promoters.

### Developmentally dynamic promoters (DDPs) analysis

In each organ, we identified promoters with dynamic temporal profiles using maSigPro, an R package designed for transcriptomic time courses [15,22]. We applied maSigPro to the absolute promoter activity data with a polynomial degree of 3. Promoters were classified as DDPs when the goodness-of-fit (R²) was at least 0.3 in a given organ.

### Transcription factor motif enrichment analysis

We used PWMEnrich [23] to identify transcription factor (TF) motif enrichment for major and minor promoters, defined as the 200 bp upstream of the transcription start site (TSS), within each category. Results were filtered to include only those with a p-value < 0.01.

### Identification of common and organ-specific DDPs

To classify DDPs, we compared the DDP corresponding gene names of each tissue against those of other organs. DDPs present in at least two organs were categorized as common DDPs, whereas those unique to a single tissue were classified as organ-specific DDPs. Notably, DDPs shared exclusively between the brain and cerebellum were not classified as common DDPs due to the close functional relationship between these two organs.

### Data visualization

The boxplot, barplot, histogram, scatterplot, lineplot, dotplot and donut plot were produced with ggplot2 (v3.3.2; https://ggplot2.tidyverse.org). Heatmaps displaying gene expression changes were created using the pheatmap package (v1.0.12; https://cran.r-project.org/web/packages/pheatmap/index.html). Sankey plot is generated with R package networkD3 (v0.4; https://CRAN.R-project.org/package=networkD3), while t-SNE plot is generated with R package Rtsne (v0.16; https://github.com/jkrijthe/Rtsne). GO enrichment analysis is performed by R package ClusterProfile [24] in R (v 4.0.3).

## Supporting information

S1 Fig Analysis of genes corresponding to developmentally active promoters. (A) Histogram showing the distribution of gene numbers based on the number of promoters associated with each gene. (B-D) t-SNE plots representing the top 2,000 genes with the highest variance in gene activity across all RNA-seq samples: (B) all samples, (C) male samples, and (D) female samples.
(PDF)

S2 Fig Characterization of developmentally dynamic promoters in the brain. (A-H) Developmental dynamic analysis of brain RNA-seq data. The major and minor/alternative promoter pairs show eight distinct patterns along developmental time increasing, see Fig 2A-H. For each category, the upper panel shows the mean value of overall gene activity (sum of all promoter activities) changes over developmental stages. The middle panel shows the mean value of major and minor/alternative promoter activity changes over developmental stages, the shadow represents 1000 bootstrap. The bottom panel is the heatmap depicting each major or minor/alternative promoter’s activity over developmental stages.
(PDF)

S3 Fig Characterization of developmentally dynamic promoters in the cerebellum. (A-H) Developmental dynamic analysis of cerebellum RNA-seq data. The major and minor/alternative promoter pairs show eight distinct patterns along developmental time increasing, see Fig 2A-H. For each category, the upper panel shows the mean value of overall gene activity (sum of all promoter activities) changes over developmental stages. The middle panel shows the mean value of major and minor/alternative promoter activity changes over developmental stages, the shadow represents 1000 bootstrap. The bottom panel is the heatmap depicting each major or minor/alternative promoter’s activity over developmental stages.
(PDF)

S4 Fig Characterization of developmentally dynamic promoters in the heart. (A-H) Developmental dynamic analysis of heart RNA-seq data. The major and minor/alternative promoter pairs show eight distinct patterns along developmental time increasing, see Fig 2A-H. For each category, the upper panel shows the mean value of overall gene activity (sum of all promoter activities) changes over developmental stages. The middle panel shows the mean value of major and minor/alternative promoter activity changes over developmental stages, the shadow represents 1000 bootstrap. The bottom panel is the heatmap depicting each major or minor/alternative promoter’s activity over developmental stages.
(PDF)

S5 Fig Characterization of developmentally dynamic promoters in the kidney. (A-H) Developmental dynamic analysis of kidney RNA-seq data. The major and minor/alternative promoter pairs show eight distinct patterns along developmental time increasing, see Fig 2A-H. For each category, the upper panel shows the mean value of overall gene activity (sum of all promoter activities) changes over developmental stages. The middle panel shows the mean value of major and minor/alternative promoter activity changes over developmental stages, the shadow represents 1000 bootstrap. The bottom panel is the heatmap depicting each major or minor/alternative promoter’s activity over developmental stages.
(PDF)

S6 Fig Characterization of developmentally dynamic promoters in the liver. (A-H) Developmental dynamic analysis of liver RNA-seq data. The major and minor/alternative promoter pairs show eight distinct patterns along developmental time increasing, see Fig 2A-H. For each category, the upper panel shows the mean value of overall gene activity (sum of all promoter activities) changes over developmental stages. The middle panel shows the mean value of major and minor/alternative promoter activity changes over developmental stages, the shadow represents 1000 bootstrap. The bottom panel is the heatmap depicting each major or minor/alternative promoter’s activity over developmental stages.
(PDF)

S7 Fig Characterization of developmentally dynamic promoters in the ovary. (A-H) Developmental dynamic analysis of ovary RNA-seq data. The major and minor/alternative promoter pairs show eight distinct patterns along developmental time increasing, see Fig 2A-H. For each category, the upper panel shows the mean value of overall gene activity (sum of all promoter activities) changes over developmental stages. The middle panel shows the mean value of major and minor/alternative promoter activity changes over developmental stages, the shadow represents 1000 bootstrap. The bottom panel is the heatmap depicting each major or minor/alternative promoter’s activity over developmental stages.
(PDF)

S8 Fig Comparison of promoter activity between DDPs and flat promoters in each organ. (A-G) Boxplots comparing the activity levels of developmentally dynamic promoters (DDPs) and flat (developmentally unchanging) promoters in each organ. Promoter activity for each promoter is calculated using three metrics: the maximum value across all developmental stages (left panel), the mean value across all stages (middle panel), and the median value across all stages (right panel). Each boxplot displays the median (central line), the interquartile range (IQR; box boundaries), and 1.5× the IQR (whiskers). Statistical significance was assessed using two-sided nonparametric Wilcoxon rank-sum tests, with exact p-values indicated on the plots.
(PDF)

S9 Fig Transcription factor motif enrichment of major and minor/alternative DDPs. (A-H) Enrichment analysis of transcription factor (TF) motifs for major and minor/alternative promoters across different categories in each organ. The dot plot visualization displays key enrichment results, with dot size representing the -log10(p-value), indicating the statistical significance of the TF motif enrichment, and dot color representing the motif occurrence proportion, reflecting the frequency of the corresponding TF motif in the analyzed promoter regions.
(PDF)

S10 Fig Overlap of Enriched GO Terms Across Organs. (A) UpsetR plot showing the overlap of Gene Ontology (GO) terms enriched among common DDPs across different organs. Common DDPs, defined as those shared by at least two organs, are associated with fundamental biological processes. The plot highlights intersections of enriched GO terms among organ combinations, illustrating shared functional roles across tissues. (B) UpsetR plot illustrating the overlap of enriched GO terms among organ-specific DDPs. Organ-specific DDPs, unique to a single tissue, exhibit enrichment in specialized biological processes relevant to their respective organ functions. The plot emphasizes the small overlap of GO terms between tissues, reflecting the distinct regulatory roles of organ-specific DDPs in driving tissue-specific functions.
(PDF)

S11 Fig Proportions of coding and noncoding transcripts associated with alternative promoters across categories. (A-G) The bar plot displays the percentage of protein-coding and noncoding transcripts for each category of alternative promoters in each organ. The annotation of different transcripts is from Ensembl.
(PDF)

S1 Table A comprehensive list of promoter coordinates, associated genes, promoter types, and activity levels across developmental stages and tissues. (TXT)

S2 Table Developmental dynamic promoter categories across tissues. (TXT)

S3 Table Transcription factor motif enrichment results in developmental dynamic promoter categories. (TXT)

S4 Table Enriched GO terms associated with developmental dynamic promoter categories. (TXT)

## References

[1] Carninci P, Sandelin A, Lenhard B, Katayama S, Shimokawa K, Ponjavic J, et al. Genome-wide analysis of mammalian promoter architecture and evolution. Nat Genet. 2006;38(6):626–35. doi: 10.1038/ng1789 16645617

[2] Sandelin A, Carninci P, Lenhard B, Ponjavic J, Hayashizaki Y, Hume DA. Mammalian RNA polymerase II core promoters: insights from genome-wide studies. Nat Rev Genet. 2007;8(6):424–36. doi: 10.1038/nrg2026 17486122

[3] Ayoubi TA, Van De Ven WJ. Regulation of gene expression by alternative promoters. FASEB J. 1996;10(4):453–60. 8647344

[4] Bharti K, Liu W, Csermely T, Bertuzzi S, Arnheiter H. Alternative promoter use in eye development: the complex role and regulation of the transcription factor MITF. Development. 2008;135(6):1169–78. doi: 10.1242/dev.014142 18272592 PMC2276638

[5] Barrera LO, Li Z, Smith AD, Arden KC, Cavenee WK, Zhang MQ, et al. Genome-wide mapping and analysis of active promoters in mouse embryonic stem cells and adult organs. Genome Res. 2008;18(1):46–59. doi: 10.1101/gr.6654808 18042645 PMC2134779

[6] Feng G, Tong M, Xia B, Luo G-Z, Wang M, Xie D, et al. Ubiquitously expressed genes participate in cell-specific functions via alternative promoter usage. EMBO Rep. 2016;17(9):1304–13. doi: 10.15252/embr.201541476 27466324 PMC5007564

[7] Pal S, Gupta R, Kim H, Wickramasinghe P, Baubet V, Showe LC, et al. Alternative transcription exceeds alternative splicing in generating the transcriptome diversity of cerebellar development. Genome Res. 2011;21(8):1260–72. doi: 10.1101/gr.120535.111 21712398 PMC3149493

[8] Reyes A, Huber W. Alternative start and termination sites of transcription drive most transcript isoform differences across human tissues. Nucleic Acids Res. 2018;46(2):582–92. doi: 10.1093/nar/gkx1165 29202200 PMC5778607

[9] Cardoso-Moreira M, Halbert J, Valloton D, Velten B, Chen C, Shao Y, et al. Gene expression across mammalian organ development. Nature. 2019;571(7766):505–9. doi: 10.1038/s41586-019-1338-5 31243369 PMC6658352

[10] Mazin PV, Khaitovich P, Cardoso-Moreira M, Kaessmann H. Alternative splicing during mammalian organ development. Nat Genet. 2021;53(6):925–34. doi: 10.1038/s41588-021-00851-w 33941934 PMC8187152

[11] Sarropoulos I, Marin R, Cardoso-Moreira M, Kaessmann H. Developmental dynamics of lncRNAs across mammalian organs and species. Nature. 2019;571(7766):510–4. doi: 10.1038/s41586-019-1341-x 31243368 PMC6660317

[12] Harrow J, Frankish A, Gonzalez JM, Tapanari E, Diekhans M, Kokocinski F, et al. GENCODE: the reference human genome annotation for The ENCODE Project. Genome Res. 2012;22(9):1760–74. doi: 10.1101/gr.135350.111 22955987 PMC3431492

[13] Frith MC, Valen E, Krogh A, Hayashizaki Y, Carninci P, Sandelin A. A code for transcription initiation in mammalian genomes. Genome Res. 2008;18(1):1–12. doi: 10.1101/gr.6831208 18032727 PMC2134772

[14] Demircioğlu D, Cukuroglu E, Kindermans M, Nandi T, Calabrese C, Fonseca NA, et al. A Pan-cancer Transcriptome Analysis Reveals Pervasive Regulation through Alternative Promoters. Cell. 2019;178(6):1465–77.e17. doi: 10.1016/j.cell.2019.08.018 31491388

[15] Nueda MJ, Tarazona S, Conesa A. Next maSigPro: updating maSigPro bioconductor package for RNA-seq time series. Bioinformatics. 2014;30(18):2598–602. doi: 10.1093/bioinformatics/btu333 24894503 PMC4155246

[16] Neumayr C, Haberle V, Serebreni L, Karner K, Hendy O, Boija A, et al. Differential cofactor dependencies define distinct types of human enhancers. Nature. 2022;606(7913):406–13. doi: 10.1038/s41586-022-04779-x 35650434 PMC7613064

[17] Sears K, Maier JA, Sadier A, Sorensen D, Urban DJ. Timing the developmental origins of mammalian limb diversity. Genesis. 2018;56(1): 10.1002/dvg.23079 29095555

[18] Abzhanov A. von Baer’s law for the ages: lost and found principles of developmental evolution. Trends Genet. 2013;29(12):712–22. doi: 10.1016/j.tig.2013.09.004 24120296

[19] Bhardwaj V, Heyne S, Sikora K, Rabbani L, Rauer M, Kilpert F, et al. snakePipes: facilitating flexible, scalable and integrative epigenomic analysis. Bioinformatics. 2019;35(22):4757–9. doi: 10.1093/bioinformatics/btz436 31134269 PMC6853707

[20] Dobin A, Davis CA, Schlesinger F, Drenkow J, Zaleski C, Jha S, et al. STAR: ultrafast universal RNA-seq aligner. Bioinformatics. 2013;29(1):15–21. doi: 10.1093/bioinformatics/bts635 23104886 PMC3530905

[21] Love MI, Huber W, Anders S. Moderated estimation of fold change and dispersion for RNA-seq data with DESeq2. Genome Biol. 2014;15(12):550. doi: 10.1186/s13059-014-0550-8 25516281 PMC4302049

[22] Conesa A, Nueda MJ, Ferrer A, Talón M. maSigPro: a method to identify significantly differential expression profiles in time-course microarray experiments. Bioinformatics. 2006;22(9):1096–102. doi: 10.1093/bioinformatics/btl056 16481333

[23] Robert Stojnic DD. PWMEnrich. Bioconductor; 2017. doi:10.18129/B9.BIOC.PWMENRICH

[24] Yu G, Wang L-G, Han Y, He Q-Y. clusterProfiler: an R package for comparing biological themes among gene clusters. OMICS. 2012;16(5):284–7. doi: 10.1089/omi.2011.0118 22455463 PMC3339379

