# Supplementary figures and images for: Alternative promoter usage during organ development
Source: PLoS Genet. 2025 Mar 28;21(3):e1011635. doi: 10.1371/journal.pgen.1011635 (PMC11978060; doi:10.1371/journal.pgen.1011635)

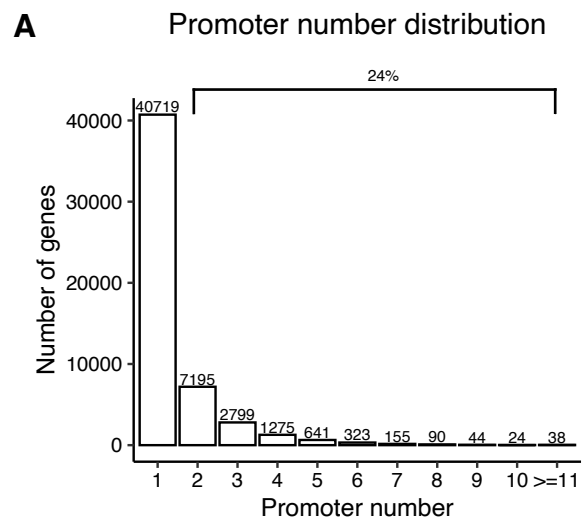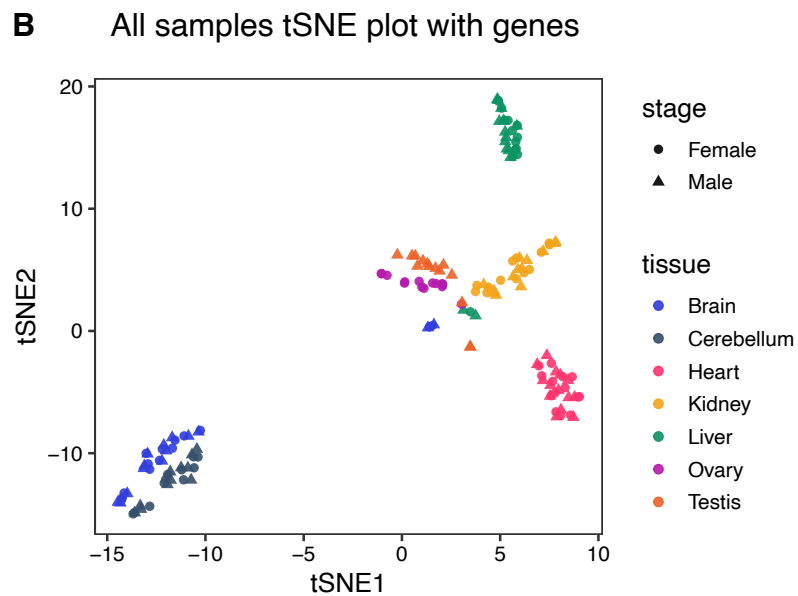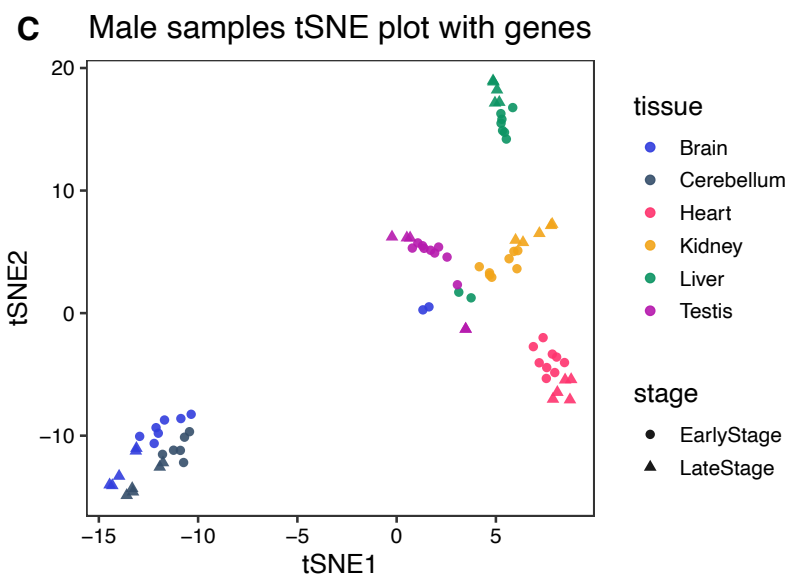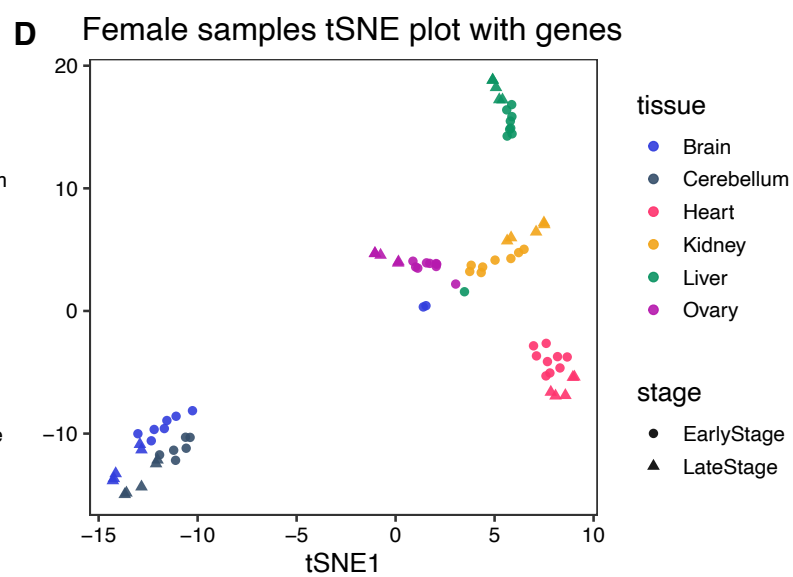

Supplement: S1 Fig — (A) Histogram showing the distribution of gene numbers based on the number of promoters associated with each gene. (B-D) t-SNE plots representing the top 2,000 genes with the highest variance in gene activity across all RNA-seq samples: (B) all samples, (C) male samples, and (D) female samples. (PDF) [file pgen.1011635.s001.pdf]

# Brain

## 1: Up regulated genes

## 2: Down regulated genes

## 3: Unchanged genes

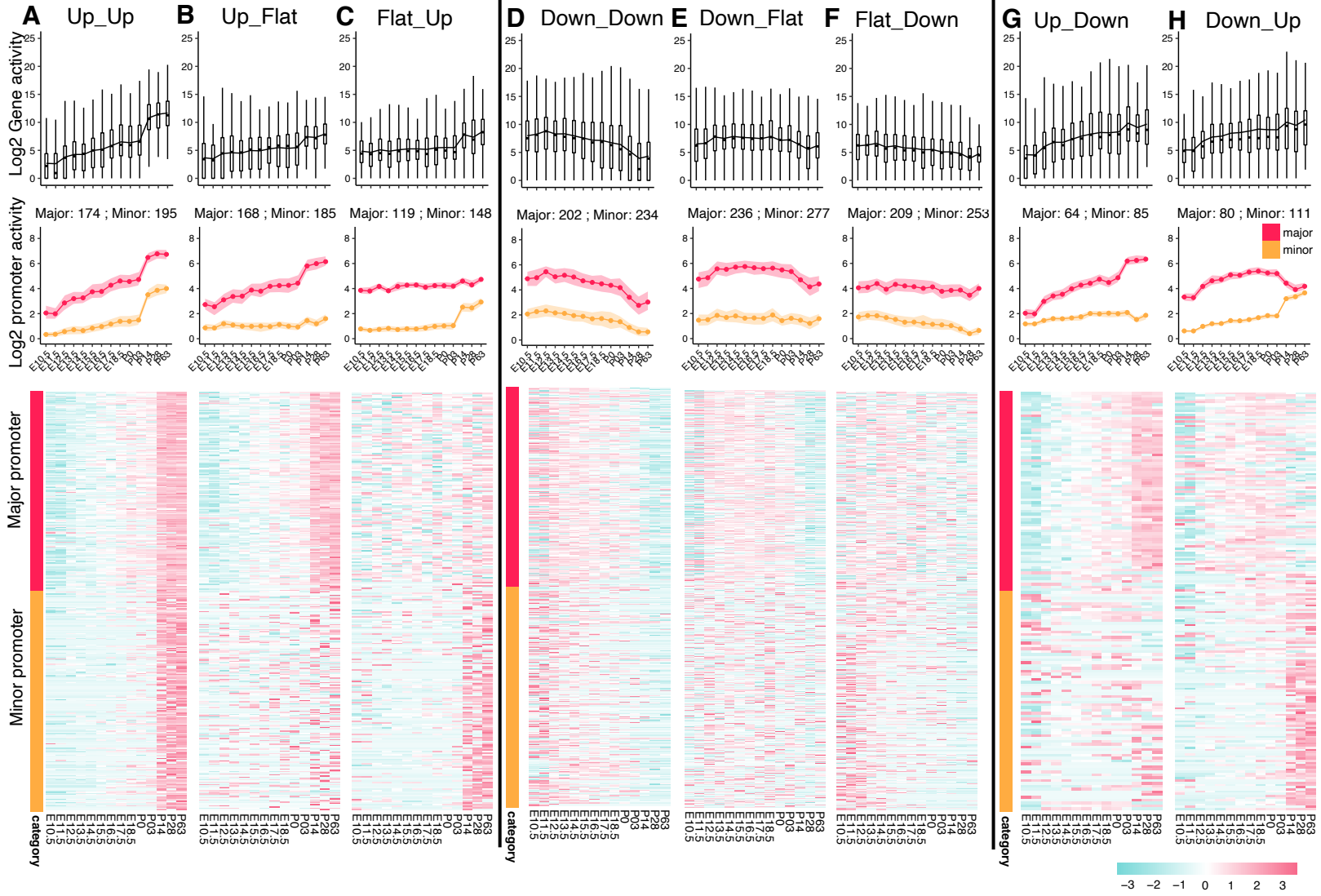

Supplement: S2 Fig — (A-H) Developmental dynamic analysis of brain RNA-seq data. The major and minor/alternative promoter pairs show eight distinct patterns along developmental time increasing, see Fig 2A-H. For each category, the upper panel shows the mean value of overall gene activity (sum of all promoter activities) changes over developmental stages. The middle panel shows the mean value of major and minor/alternative promoter activity changes over developmental stages, the shadow represents 1000 bootstrap. The bottom panel is the heatmap depicting each major or minor/alternative promoter’s activity over developmental stages. (PDF) [file pgen.1011635.s002.pdf]

## Cerebellum

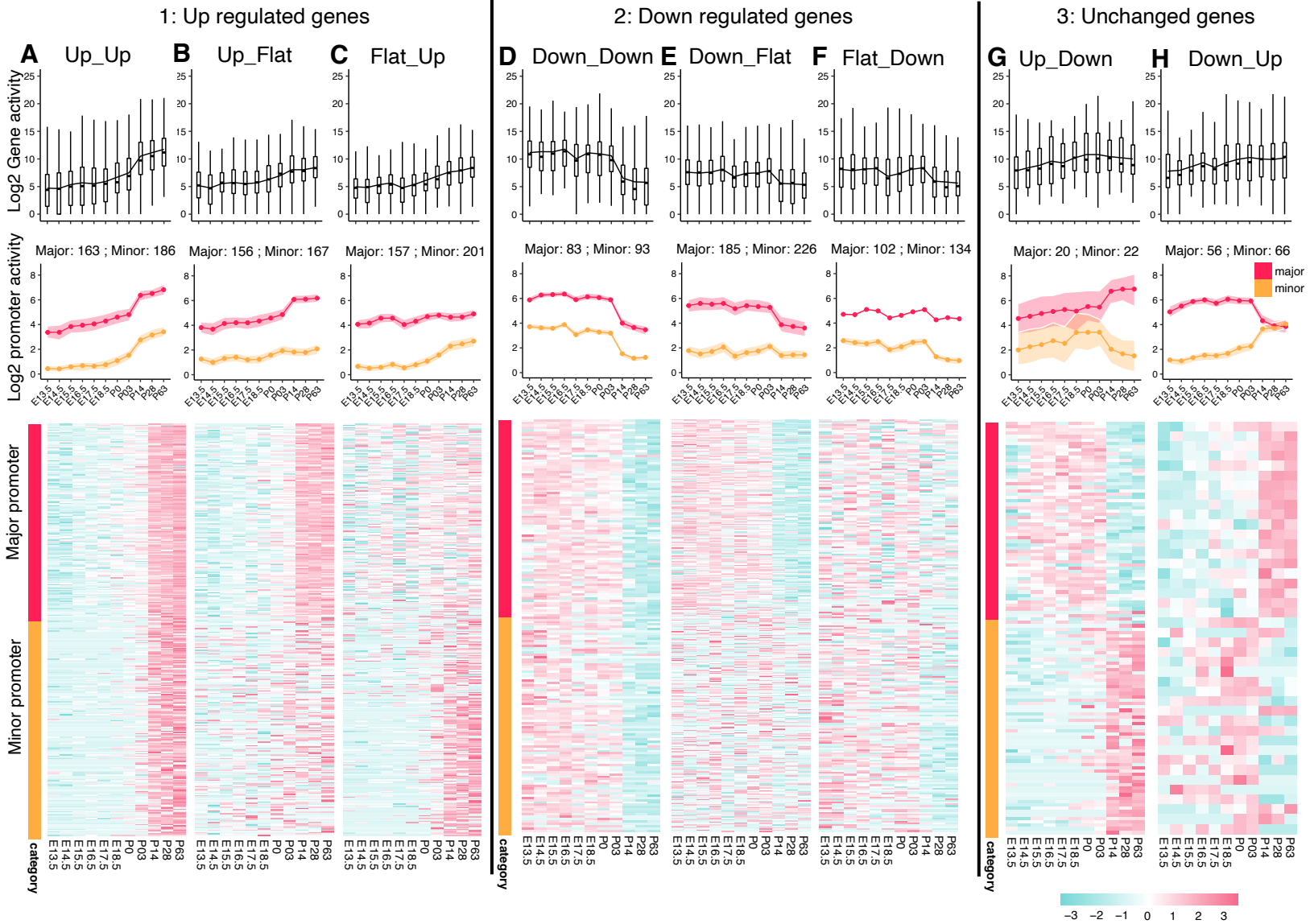

Supplement: S3 Fig — (A-H) Developmental dynamic analysis of cerebellum RNA-seq data. The major and minor/alternative promoter pairs show eight distinct patterns along developmental time increasing, see Fig 2A-H. For each category, the upper panel shows the mean value of overall gene activity (sum of all promoter activities) changes over developmental stages. The middle panel shows the mean value of major and minor/alternative promoter activity changes over developmental stages, the shadow represents 1000 bootstrap. The bottom panel is the heatmap depicting each major or minor/alternative promoter’s activity over developmental stages. (PDF) [file pgen.1011635.s003.pdf]

## Heart

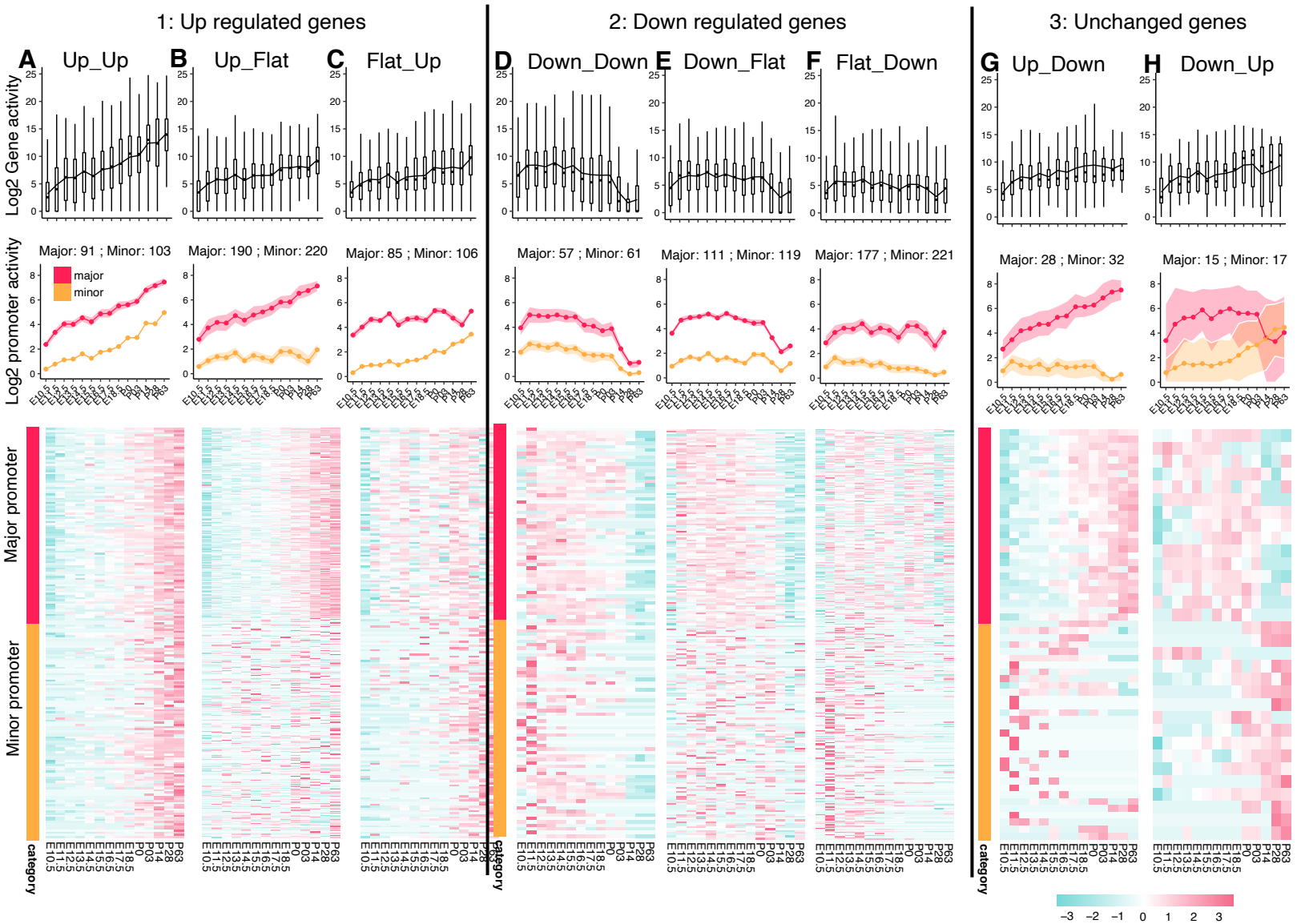

S4 Figure

Supplement: S4 Fig — (A-H) Developmental dynamic analysis of heart RNA-seq data. The major and minor/alternative promoter pairs show eight distinct patterns along developmental time increasing, see Fig 2A-H. For each category, the upper panel shows the mean value of overall gene activity (sum of all promoter activities) changes over developmental stages. The middle panel shows the mean value of major and minor/alternative promoter activity changes over developmental stages, the shadow represents 1000 bootstrap. The bottom panel is the heatmap depicting each major or minor/alternative promoter’s activity over developmental stages. (PDF) [file pgen.1011635.s004.pdf]

## Kidney

### 1: Up regulated genes

## 2: Down regulated genes

### 3: Unchanged genes

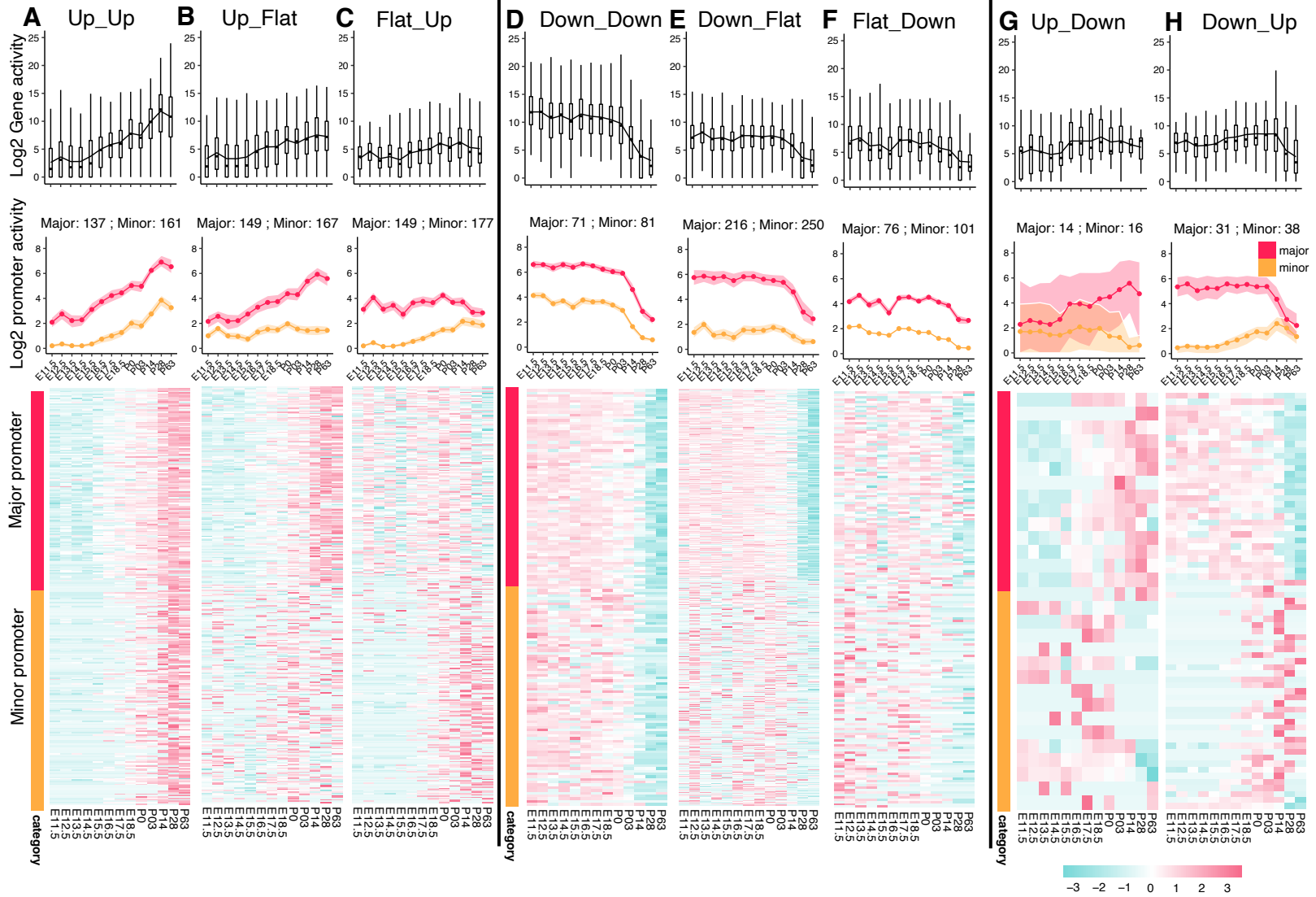

S5 Figure

Supplement: S5 Fig — (A-H) Developmental dynamic analysis of kidney RNA-seq data. The major and minor/alternative promoter pairs show eight distinct patterns along developmental time increasing, see Fig 2A-H. For each category, the upper panel shows the mean value of overall gene activity (sum of all promoter activities) changes over developmental stages. The middle panel shows the mean value of major and minor/alternative promoter activity changes over developmental stages, the shadow represents 1000 bootstrap. The bottom panel is the heatmap depicting each major or minor/alternative promoter’s activity over developmental stages. (PDF) [file pgen.1011635.s005.pdf]

# Liver

## 1: Up regulated genes

## 2: Down regulated genes

## 3: Unchanged genes

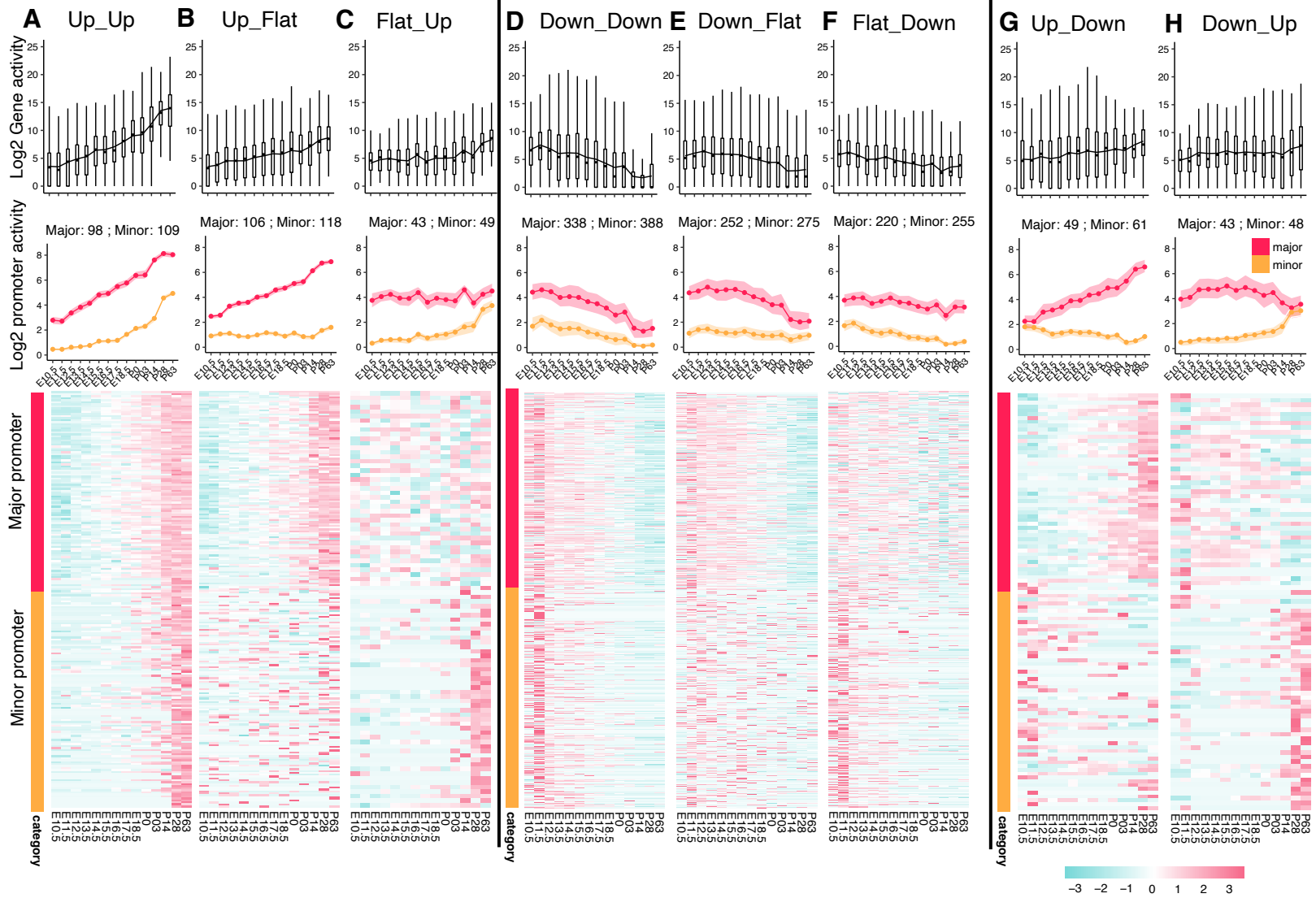

Supplement: S6 Fig — (A-H) Developmental dynamic analysis of liver RNA-seq data. The major and minor/alternative promoter pairs show eight distinct patterns along developmental time increasing, see Fig 2A-H. For each category, the upper panel shows the mean value of overall gene activity (sum of all promoter activities) changes over developmental stages. The middle panel shows the mean value of major and minor/alternative promoter activity changes over developmental stages, the shadow represents 1000 bootstrap. The bottom panel is the heatmap depicting each major or minor/alternative promoter’s activity over developmental stages. (PDF) [file pgen.1011635.s006.pdf]

Ovary

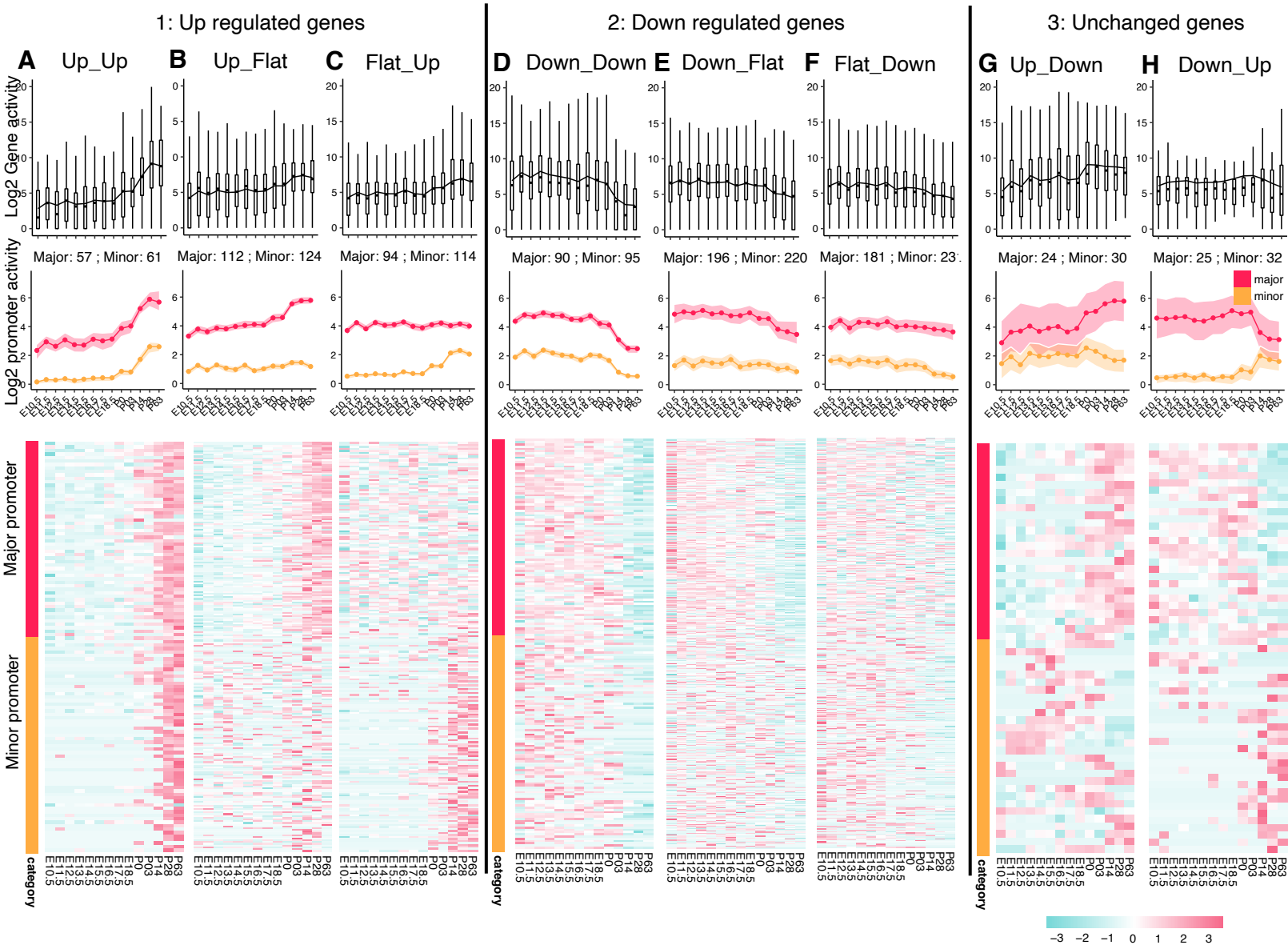

Supplement: S7 Fig — (A-H) Developmental dynamic analysis of ovary RNA-seq data. The major and minor/alternative promoter pairs show eight distinct patterns along developmental time increasing, see Fig 2A-H. For each category, the upper panel shows the mean value of overall gene activity (sum of all promoter activities) changes over developmental stages. The middle panel shows the mean value of major and minor/alternative promoter activity changes over developmental stages, the shadow represents 1000 bootstrap. The bottom panel is the heatmap depicting each major or minor/alternative promoter’s activity over developmental stages. (PDF) [file pgen.1011635.s007.pdf]

**G**

Median value

## Ovary

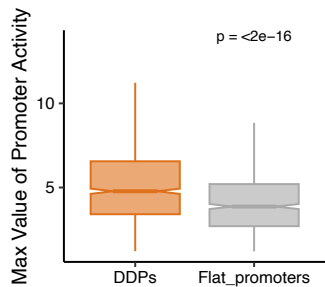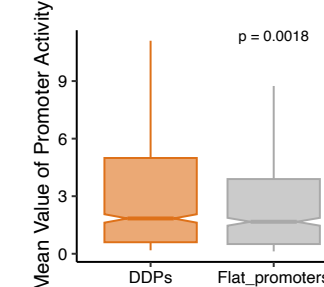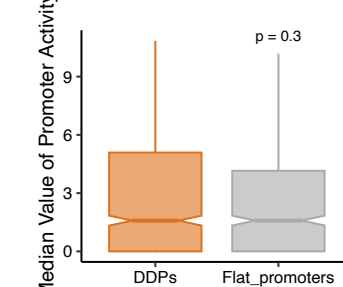

S8 Figure

Supplement: S8 Fig — (A-G) Boxplots comparing the activity levels of developmentally dynamic promoters (DDPs) and flat (developmentally unchanging) promoters in each organ. Promoter activity for each promoter is calculated using three metrics: the maximum value across all developmental stages (left panel), the mean value across all stages (middle panel), and the median value across all stages (right panel). Each boxplot displays the median (central line), the interquartile range (IQR; box boundaries), and 1.5× the IQR (whiskers). Statistical significance was assessed using two-sided nonparametric Wilcoxon rank-sum tests, with exact p-values indicated on the plots. (PDF) [file pgen.1011635.s008.pdf]

### 3: Unchanged genes

## H Down\_Up

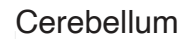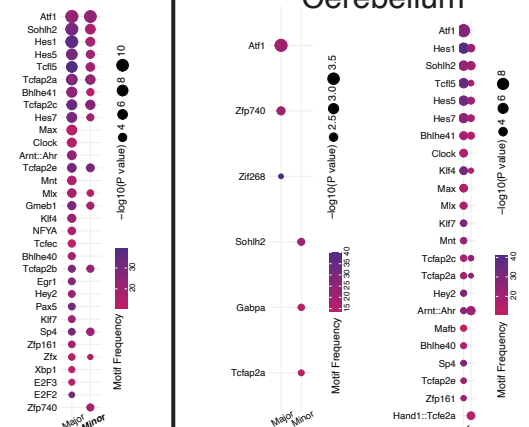

## Kidney

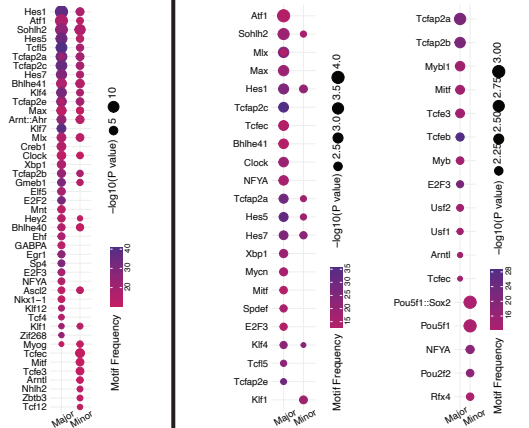

Ovary

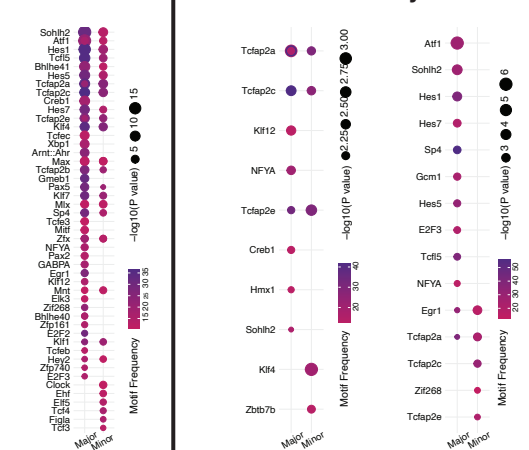

Supplement: S9 Fig — (A-H) Enrichment analysis of transcription factor (TF) motifs for major and minor/alternative promoters across different categories in each organ. The dot plot visualization displays key enrichment results, with dot size representing the -log10(p-value), indicating the statistical significance of the TF motif enrichment, and dot color representing the motif occurrence proportion, reflecting the frequency of the corresponding TF motif in the analyzed promoter regions. (PDF) [file pgen.1011635.s009.pdf]

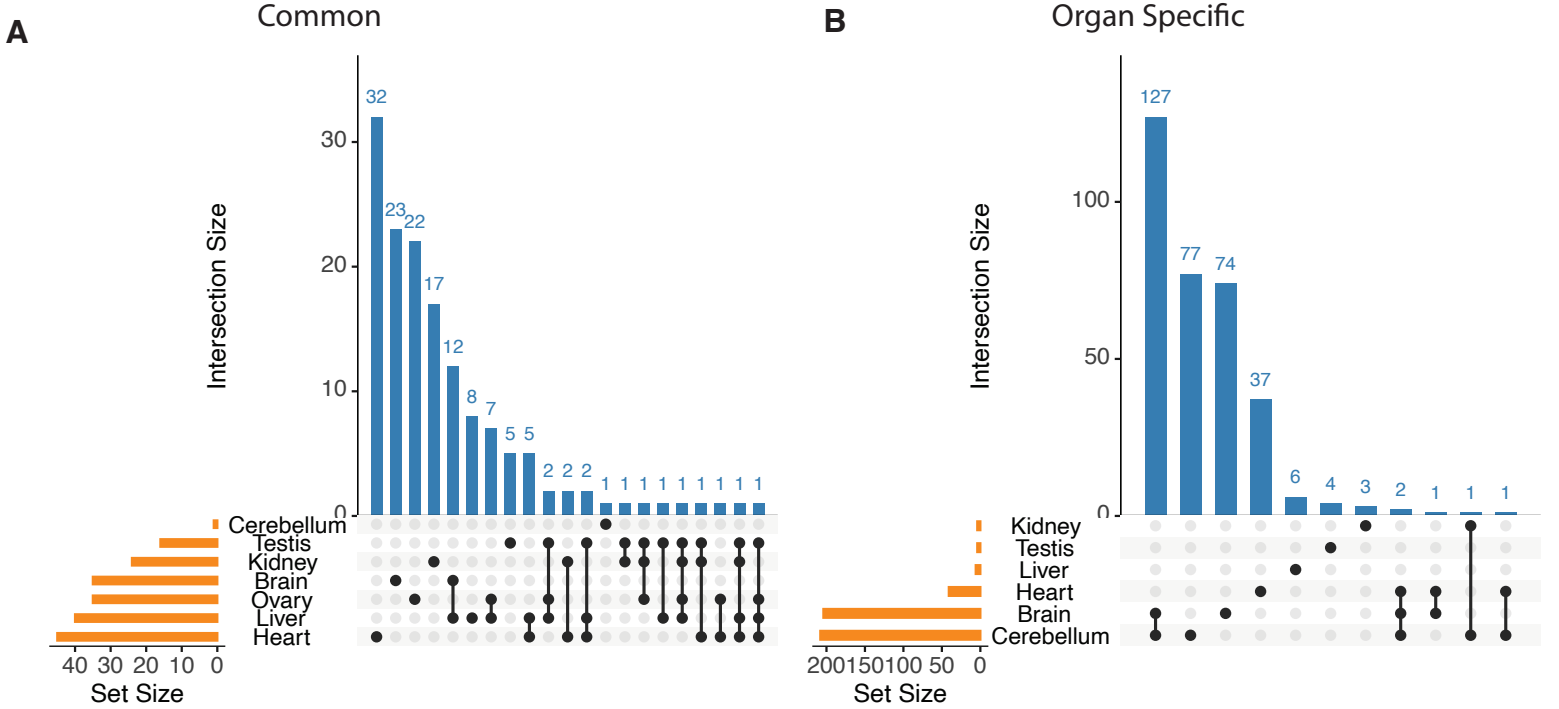

S10 Figure

Supplement: S10 Fig — (A) UpsetR plot showing the overlap of Gene Ontology (GO) terms enriched among common DDPs across different organs. Common DDPs, defined as those shared by at least two organs, are associated with fundamental biological processes. The plot highlights intersections of enriched GO terms among organ combinations, illustrating shared functional roles across tissues. (B) UpsetR plot illustrating the overlap of enriched GO terms among organ-specific DDPs. Organ-specific DDPs, unique to a single tissue, exhibit enrichment in specialized biological processes relevant to their respective organ functions. The plot emphasizes the small overlap of GO terms between tissues, reflecting the distinct regulatory roles of organ-specific DDPs in driving tissue-specific functions. (PDF) [file pgen.1011635.s010.pdf]

A

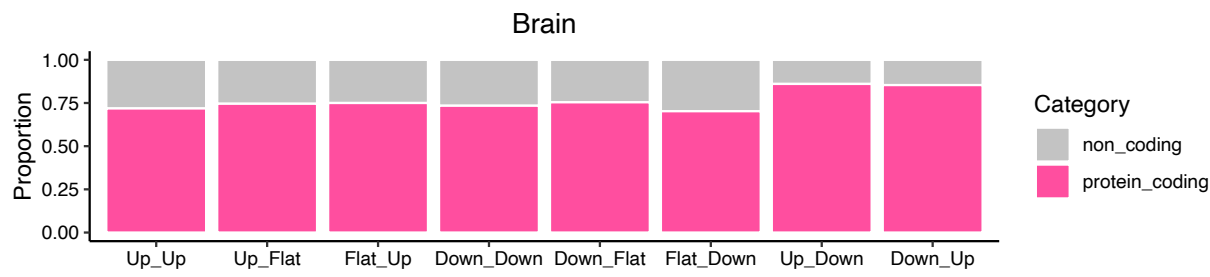

B

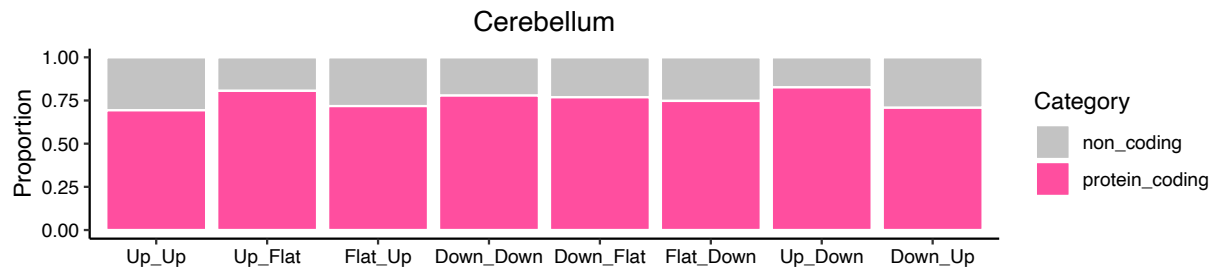

C

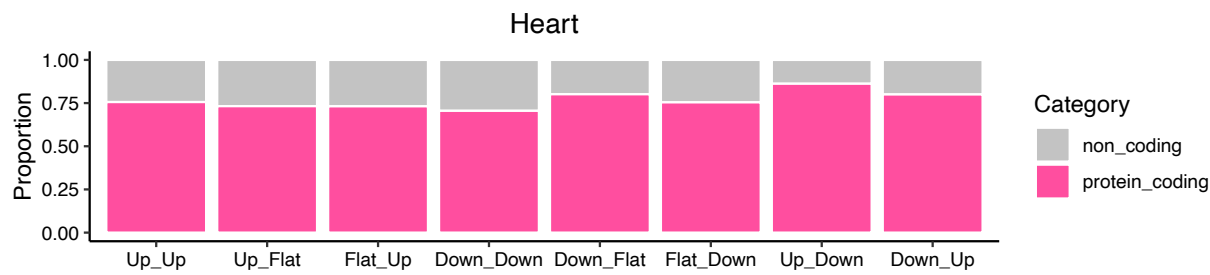

D

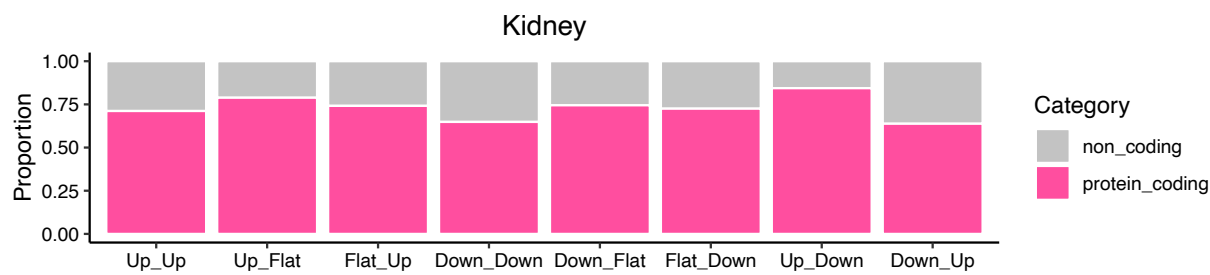

E

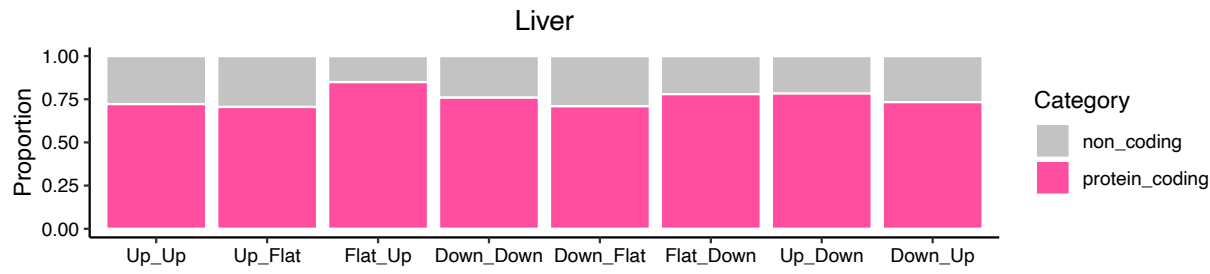

F

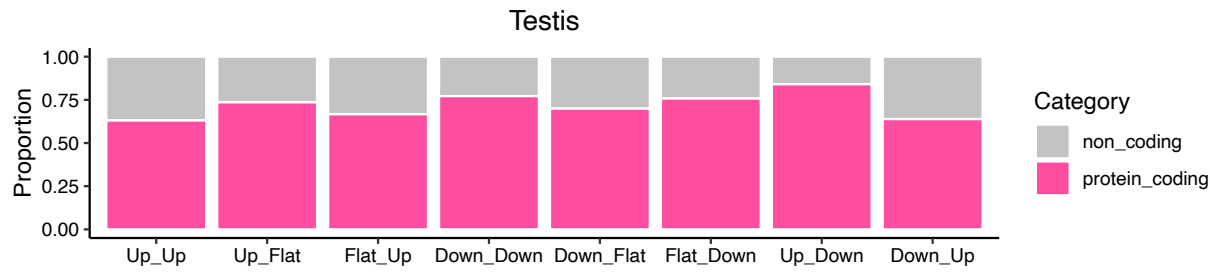

G

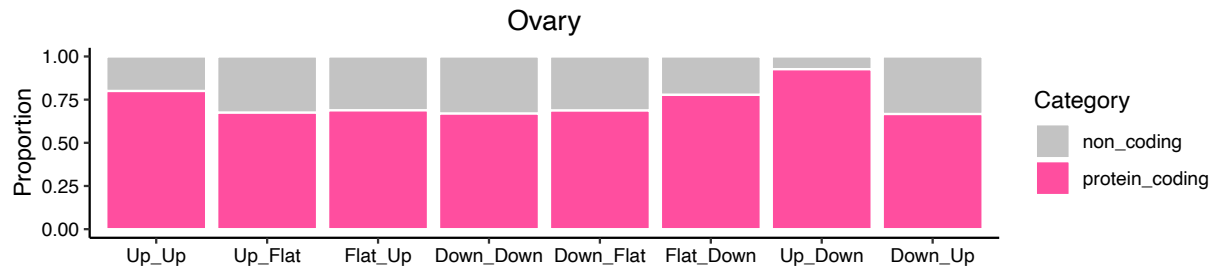

S11 Figure

Supplement: S11 Fig — (A-G) The bar plot displays the percentage of protein-coding and noncoding transcripts for each category of alternative promoters in each organ. The annotation of different transcripts is from Ensembl. (PDF) [file pgen.1011635.s011.pdf]
